# Identification of a non-host semiochemical from tick-resistant donkeys (*Equus asinus*) against *Amblyomma sculptum* ticks

**DOI:** 10.1016/j.ttbdis.2019.02.006

**Published:** 2019-04

**Authors:** Lorena Lopes Ferreira, André Lucio Franceschini Sarria, Jaires Gomes de Oliveira Filho, Fernanda de Oliveira de Silva, Stephen J. Powers, John C. Caulfield, John A. Pickett, Michael A. Birkett, Lígia Miranda Ferreira Borges

**Affiliations:** a Escola de Veterinária e Zootecnia, Universidade Federal de Goiás, Campus Samambaia, Avenida Esperança, s/n, Campus Universitário, CEP: 74690-900, Goiânia, Goiás, Brazil; b Rothamsted Research, West Common, Harpenden, Hertfordshire AL5 2JQ, United Kingdom; c Instituto de Patologia Tropical e Saúde Pública, Universidade Federal de Goiás, Rua 235 s/n, Setor Universitário, CEP: 74605050, Goiânia, Goiás, Brazil

**Keywords:** *Amblyomma cajennense* sensu lato, Donkey, Non-host, *(E)*-2-octenal, Repellent, Allomone

## Abstract

*Amblyomma sculptum* is a tick affecting animal and human health across Argentina, Bolivia, Paraguay and Brazil. Donkeys, *Equus asinus*, are known to be resistant to *A. sculptum*, suggesting that they can produce non-host tick semiochemicals (allomones), as already demonstrated for some other vertebrate host/pest interactions, whereas horses, *Equus caballus*, are considered as susceptible hosts. In this study, we tested the hypothesis that donkeys produce natural repellents against *A. sculptum*, by collecting sebum from donkeys and horses, collecting the odour from sebum extracts, and identifying donkey-specific volatile compounds by gas chromatography (GC) and coupled GC-mass spectrometry (GC–MS). From the complex collected blends, five main compounds were identified in both species. Hexanal, heptanal and *(E)*-2-decenal were found predominantly in donkey extracts, whilst ethyl octanoate and ethyl decanoate were found predominantly in horse extracts. One compound, (*E*)-2-octenal, was detected exclusively in donkey extracts. In Y-tube olfactometer bioassays 36 different *A. sculptum* nymphs were tested for each extract, compound and concentration. The dry sebum extracts and the compounds identified in both species induced neither attraction nor repellency. Only (*E*)-2-octenal, the donkey-specific compound, displayed repellency, with more nymphs preferring the arm containing the solvent control when the compound was presented in the test arm across four concentrations tested (p < 0.05, Chi-square test). A combination of a tick attractant (ammonia) and *(E)*-2-octenal at 0.25 M also resulted in preference for the control arm (p < 0.05, Chi-square test). The use of semiochemicals (allomones) identified from less-preferred hosts in tick management has been successful for repelling brown dog ticks, *Rhipicephalus sanguineus* sensu lato from dog hosts. These results indicate that (*E*)-2-octenal could be used similarly to interfere in tick host location and be developed for use in reducing *A. sculptum* numbers on animal and human hosts.

## 1 Introduction

The tick species complex *Amblyomma cajennense* (Fabricius, 1787) (Ixodida: Ixodidae) (Nava et al., 2014) is distributed across the New World, with a wide range of hosts including mammals, birds and reptiles (Barros-Battesti et al., 2006). *Amblyomma sculptum* (Berlese, 1888), that belongs to *A. cajennense* complex, is distributed across Argentina, Bolivia, Paraguay and Brazil (Nava et al., 2014), and is considered the most important vector of *Rickettsia rickettsii* (Rickettsiales: Rickettsiaceae), the causative agent of Brazilian spotted fever (BSF) in human beings. Individuals infected with this bacterium have a high fatality rate that can reach up to 85% (Araujo et al., 2015). Horses and capybaras are preferred hosts for *A. sculptum* and help maintain tick populations in rural, grass plains and rainforest environments, where those hosts are mainly found. Furthermore, capybaras are known to sustain the BSF epidemiological chain (Labruna, 2009). Parasitism by *A. sculptum* causes blood spoliation and skin damage on horses, reducing their market value, and increases production costs with treatment to reduce injurious tick bites and consequently putative transmission of tick-borne disease agents (Barros Battesti et al., 2006). The role of *A. sculptum* as a vector of *Theileria equi*, the causative agent of equine piroplasmosis, in horses remains controversial (Kerber et al., 2009; Peckle et al., 2017; Ribeiro et al., 2011). For management of *A. sculptum* populations, there are no reports to date of *A. sculptum* resistance against acaricides. However due to its low host specificity, heteroxenous life cycle and wide distribution, it is difficult to manage this tick (Cançado et al., 2017; Lopes et al., 1998; Vieira et al., 2004). The dissemination of vectors, and hence pathogens is rising due to climate change, including increasing global temperature, mobility of animals and goods, resulting in greater infection in human beings and animals. Therefore, the development of new effective tools, *via* different modes of action, to manage arthropod pests is a major challenge (Pickett et al., 2010).

The critical role of volatile semiochemicals (naturally-occurring behaviour modifying chemicals) in haematophagous arthropod ecology has been well established in recent years (Logan and Birkett, 2007; Pickett et al., 2010). Ticks use such cues to locate suitable hosts, shelters and mates. Recently, it was reported for the first time that semiochemicals play an important role in selection of suitable hosts by ticks (Borges et al., 2015), with less-preferred beagle hosts producing volatile cues 2-hexanone and benzaldehyde that act as a repellent allomone against the brown dog tick, *Rhipicephalus sanguineus* sensu lato. Oliveira Filho et al. (2017) demonstrated that a slow release formulation of those compounds decreases *R. sanguineus* s. l. load on susceptible dogs exposed in an artificially infested environment. For tsetse flies, *Glossina morsitans,* cattle are the preferred hosts, and repellents produced by non-hosts such as the waterbuck *Kobus defassa* have been shown to reduce tsetse fly infestations on cattle. Using the non-host-produced repellents, the incidence of trypanosomosis in cattle was reduced by 90% (Bett and Saini, 2015; Gikonyo et al., 2003; Saini et al., 2017). The odour from the uropygial gland of ducks can repel the red poultry mite, *Dermanyssus gallinae* (Pageat, 2005).

Donkeys, *Equus asinus*, are known to show characteristics of a non-host to *A. sculptum*. For example, when compared to horses, *E. caballus*, donkeys are less parasitized (Falce et al., 1983) and ticks that have fed on them have impairment in their development, compared to ticks that have fed on horses (Castagnolli et al., 2003). Sensitized donkeys maintain a strong resistance to *A. sculptum* infestations when compared to horses in the same conditions (Castagnolli et al., 2003). When evaluating the cutaneous reaction of the ears of donkeys and horses injected with *A. sculptum* extract, Szabó et al. (2004) observed less hypersensitivity in donkeys than in horses, suggesting that donkeys are more resistant to *A. sculptum*. In a study of serologic evidence for infection by *R. rickettsii*, an indirect immunofluorescence assay of blood samples of donkeys and horses detected antibodies reactive with *R. rickettsii* only in horse sera. The authors affirm that this result could be related to a higher resistance of donkeys against infestation by *A. sculptum* (Horta et al., 2004). Our hypothesis is that donkeys, being a non-host to *A. sculptum*, produce natural repellents against this tick species. Given the importance of *A. sculptum* to human and animal health, we proposed to test this hypothesis by identifying donkey-specific odours and conducting behavioural bioassays with identified compounds, with the aim of providing new repellents for *A. sculptum* management.

## 2 Materials and methods

The work was developed jointly by two institutions. The tick colony, sebum extraction and olfactometer tests were done at “Laboratório de Ecologia Química de Carrapatos”, “Escola de Veterinária e Zootecnia”, “Universidade Federal de Goiás” (Goiânia, Goiás – Brazil). Semiochemical collections and chemical analysis were undertaken at Rothamsted Research, United Kingdom.

### 2.1 Ethical statement and animal care

The use of animals (rabbits, donkeys and horses) was approved by the Committee on Ethical Animal Use of the Federal University of Goiás (CEUA/UFG, protocol number 024/2014). *A. sculptum* engorged females were collected from naturally infested horses from the municipality of Goiânia, Goiás, Brazil (16° 40′ 43″ S 49° 15′ 14″ W), to establish a colony that was maintained on naive rabbits (*Oryctolagus cuniculus*) from four to six months and both sexes. The rabbits were infested once with a tick stage, being maximum of 20 adults, or 100 nymphs or 700 larvae and after infestations were euthanized according with CEUA recommendations. During the infestations the rabbits were examined daily and none showed symptoms of damage due to tick parasitism. Semiochemicals were collected from 10 adult horses (*E. caballus*) and 10 donkeys (*E. asinus*) of mixed sex and aging between one to four years. All horses and donkeys were kept to pasture with water *ad libitum* being handled in accordance with the owner's handling practices.

### 2.2 Tick colony

Engorged females were placed in an acclimatized-chamber (Eletrolab, São Paulo, Brazil) at 27 ± 1 °C, RH > 80%, no light cycle, to obtain eggs. Egg masses obtained was kept in a plastic syringe with the top cut and capped with a cotton wad within the chamber at the same conditions mentioned above until the larval hatching was completed. Naïve rabbits with a chamber that was glued onto each animals shaved back were used to feed larvae (Louly et al., 2009). Engorged larvae were collected from the rabbits and maintained under the same conditions as unfed larvae until nymphal ecdysis. Unfed nymphs (30 to 50 days after ecdysis) were used in the bioassays. Nymphs were chosen because it is the stage associated with *R. rickettsii* transmission (Soares et al., 2012). To maintain the colony some nymphs were fed on rabbits and conditioned on the chamber until ecdysis as mentioned for larvae. Adults were also fed on the rabbits to obtain engorged females, and, whenever, necessary new engorged females were collected from naturally infested horses.

### 2.3 Sebum collection and extraction

Samples of skin sebum were collected from donkeys and horses using sterile cotton pads. Briefly, one side of a sterile medical cotton pad (5.5 cm diameter × 0.5 cm) was sprayed with aqueous ethanol (dissolved 1:2 in MilliQ water), then rubbed inside of a randomly chosen ear of the animal ten times using the moistened side of the pad. The pad containing the extract was placed into an empty glass vial (5 mL). Dichloromethane (DCM, 3 mL) was added to the vial and then the vial was sonicated (Ultrasonic cell disrupter – Unique, São Paulo, Brazil) for 10 min. The DCM extract was transferred using a glass pipette to a pre-weighed vial, and then evaporated to dryness under a gentle stream of nitrogen gas (Air Liquid Brazil Ltd, São Paulo, Brazil). The dried sample was sealed and stored at −20 °C until semiochemical collection and subsequent olfactometer tests.

### 2.4 Semiochemical collection from sebum material and chemical analysis

Vials containing the dry sebum were enclosed in a glass vessel (100 mL). Air was pumped through an activated charcoal filter into the vessel (1 L/min) and was then drawn (500 cc/min) into tubes containing the adsorbent Porapak Q (50 mg). After 24 h, volatiles collected on the Porapak Q were eluted with 750 μl of redistilled diethyl ether and concentrated at 100 μL under a gentle flow of nitrogen. The samples were then stored at −20 °C until analysis.

Three samples (1 μL) from each species (three separate animals per species) were randomly selected and analysed on an Agilent 6890 A GC (Agilent Technologies, Santa Clara, California, USA), equipped with a cool column injector, flame ionization detector (FID), and a DB-1 capillary GC column (50 m × 0.32 mm i.d. DB-1 x 0.52 μm film thickness, J & W Scientific). Hydrogen was the carrier gas. The oven temperature was maintained at 30 °C for 0.1 min, then programmed to increase at 10 °C min^−1^ until 250 °C, and then held for 38 min. For coupled GC–MS analysis (Waters Autospec Ultima, Manchester, UK), a DB-1 capillary GC column (50 m × 0.32 mm i.d. × 0.52 μm film thickness), equipped with a cool on-column injector was used. Ionization was accomplished by electron impact at 70 eV, 250 °C. The oven temperature was maintained at 30 °C for five minutes and then programmed to increase at a rate of 5 °C min^−1^ until 250 °C. The carrier gas was helium. Tentative identifications were made by comparing mass spectra with mass spectral databases, Kovats’ Index values and GC peak enhancement on two GC columns of differing polarity (DB-1, DB-WAX), using authentic samples of chemicals (see below).

### 2.5 Chemicals

Ethyl octanoate (98%, CAS 106-32-1), ethyl decanoate (98%, CAS 11-38-3), hexanal (98%, CAS 66-25-1), heptanal (95%, CAS 111-71-7), *(E)*-2-octenal (95%, CAS 2548-87-0), *(E)*-2-decenal (95%, CAS 3913-81-3) and dichloromethane (DCM) bi-distillate were purchased from Sigma-Aldrich, Missouri, USA. Ammonium hydroxide (P.A., 53,480) was purchased from Sigma-Aldrich, Seelze, Germany.

### 2.6 Estimation of the quantity of (*E*)-2-octenal produced by donkeys

As the primary chemical of interest, the concentration of *(E)*-2-octenal in the donkey sebum extracts was calculated using an external standard approach. Primary stock standard solutions of *(E)*-2-octenal (Aldrich Chemical Co. Ltd., Dorset, UK) at a concentration of 846 μg mL^−1^ were prepared using DCM bi-distillate (Sigma-Aldrich Co., St. Louis, Missouri, USA) as solvent. Stock solutions were diluted serially to reach a concentration of 33.84 μg mL^−1^, which was used to produce five different concentrations of *(E)*-2-octenal (25.380, 16.920, 8.460, 3.384, and 1.135 μg mL^−1^). Each final concentration was analysed in triplicate. One μl of each solution was injected and analyzed by GC-FID using the same method as detailed in the previous section. This method was adopted to account for technical variation in the calibration procedure.

### 2.7 Olfactometer bioassay

Olfactometer assays were performed using methodology adapted from Carr et al. (2013) and Borges et al. (2015). An acrylic Y-tube olfactometer was used (31 cm overall length; 13 cm for each arm, 18 cm for the stem, and a removable cap). Each arm was connected to a kitasato (odor jar) *via* a flexible polyvinyl chloride (PVC) hose (1/4 × 3/8 × 1/16 mm, Nalgene, São Paulo, Brazil). At the end of each kitasato, a fluxometer (Parker-P3A, São Paulo, Brazil) with a charcoal filter was attached and set at 0.1 L/min. The airflow was generated by a sprinkler (Diapump / Fanem, São Paulo, Brazil) connected to the opening of the olfactometer stem by a PVC hose. The vacuum was kept outside of the test room to remove the gases. To verify if the Y-tube olfactometer could be used successfully, hydrochloric acid and ammonium hydroxide (1:1) was mixed to produce a white smoke and released in both arms of the olfactometer confirming the odor plume. After that, the attraction of nymphs to synthetic air containing 5% CO_2_ (White Martins) was verified. To evaluate the compounds, one piece (1 x 4 cm) of filter paper (Whatman qualitative, number 1, Maidstone, UK) was treated with 11 μL of test substance (odor, or synthetic substance) to compare against the solvent. After treatment, the papers were dried under a fume hood (Permution, E. J. Krieger e Cia, Paraná, Brazil) for one minute before use in the bioassay. The paper was conditioned in the odor jar and the nymphs were released individually. Every five minutes, the arms were inverted and the olfactometer cleaned with ethanol (95%) and new treated papers were used. For each odor, compound/concentration, 36 different unfed nymphs were used, testing each compound and concentration in order. Each nymph was observed until an arm was chosen. If the nymph did not choose any arm in two minutes, it was tested one more time. If the nymph failed to choose one arm, it was discarded and replaced by another nymph. The following experiments were carried out: a) DCM (negative control) *vs.* blank, DCM *vs.* horse (individual dry sebum extract) and DCM *vs.* donkey odour (dry sebum extract) b) hexanal, heptanal, ethyl octanoate, ethyl decanoate, *(E)*-2-decenal and *(E)*-2-octenal, tested individually across four concentrations (1.0 M, 0.50 M, 0.25 M and 0.125 M) *vs*. DCM c) *(E*)-2-octenal at decreasing concentrations (at 0.0625 M) until no behavioural effect was observed *vs*. DCM d) ammonia, in the form of ammonium hydroxide (0.25 M) e) ammonia (0.25 M) + ***(****E)*-2-octenal **(**0.125 M) *vs.* DCM f) ammonia (0.25 M) + *(E)*-2-octenal **(**0.25 M) *vs.* DCM. Ammonia is a host related compound and has been shown to be attractive to *A. sculptum* nymphs (Ferreira, 2017). For the tests with ammonium hydroxide and *(E)*-2-octenal together, two filter papers were used, with 11 μL of each substance in the same odour jar. To standardize the odours used in dry sebum extracts, the lowest weight obtained in the extractions was verified (0.017 g), and 200 μl of DCM distillate (being the minimum volume needed for the olfactometer tests), was added. This proportion was maintained for all the other dry sebum extracts.

### 2.8 Statistical analysis

The calibration assay of *(E)*-2-octenal was determined by linear regression analysis with the chromatographic areas (mAU) obtained from the GC (Fig S1, Supplementary Information) being the response variable, *y*, and the corresponding concentrations injected to secure those areas, of *(E)*-2-octenal (μg mL^−1^), being the explanatory variable, *x*. Using the range of concentration of the compound, the linear regression model was *y* = *c* + *b*x, where *c* is the intercept and *b* is the slope of the fitted line. The strength of the relationship was judged in terms of the proportion of variation explained, R^2^, and the significance (p < 0.05) of the F-test from the analysis of variance (ANOVA) accompanying the regression (Table S1, Supplementary Information). Evidence of any curvature was assessed by adding a quadratic term in concentration into the linear model and testing the improvement in fit (F-test). The model was fitted using ordinary least squares, which provides estimates of the parameters *c* and *b* along with standard errors. The linear formulae obtained from this analysis were used to determine the concentration of each compound in each sample, by substituting in the values of areas, *y*, for the samples, to calculate the corresponding concentrations, *x*. The limit of detection (LOD) and the limit of quantification (LOQ) were determined in accordance with the linear regression, and are expressed as: LOD = 3.3×SE(*c*)/*b* and LOQ = 10×SE(*c*)/*b* where: SE(*c*) is the standard error of the intercept and *b* is the slope of the regression line. For count data from the olfactometer assays, as each set of 36 ticks was done sequentially for concentrations per test compound, the Chi-square test on one degree of freedom (df) with Yates’ correction (Yates, 1934) was performed, taking the significance level of p < 0.05.

## 3 Results

### 3.1 GC and GC–MS analysis

**Fig. 1:**
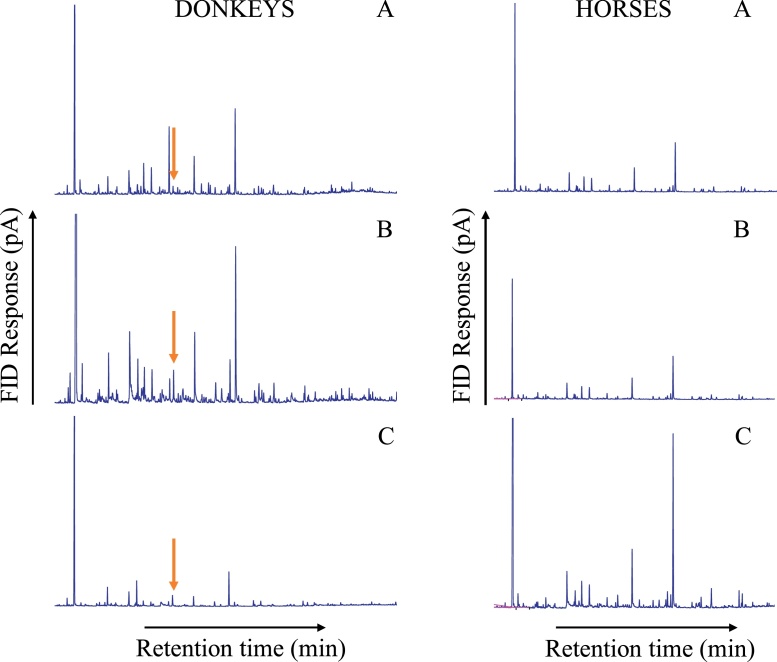
Typical gas chromatograph analysis from donkey (A, B, C) and horse (D, E, F) chemical extractions using protocol described in Materials and Methods. Arrows show the peak corresponding to (*E*)-2-octenal which is found almost exclusively in donkeys.

Gas chromatography (GC) analysis of volatile organic compound (VOC) extracts collected from horse and donkey sebum by air entrainment revealed the presence of a large number of compounds (Fig. 1). Comparison of extracts between horses and donkeys revealed the presence of four compounds in higher amounts in donkey extracts than in horse extracts, which were tentatively identified by coupled GC-mass spectrometry (GC–MS) and confirmed by GC peak enhancement as hexanal, heptanal, *(E)*-2-decenal and *(E)*-2-octenal. Minor quantities of the first three compounds were found in horse VOCs, but for the latter compound, none was detected. Furthermore, two compounds, ethyl decanoate and ethyl octanoate, were mostly present in horse extracts. The concentration of *(E)*-2-octenal in the sebum from donkeys was 0.946 μg mL^−1^ (SE 0.090) in donkey 1, 4.940 (SE 0.076) μg ml^−1^ in donkey 2 and 1.920 (SE 0.086) μg mL^−1^ in donkey 3. The mean concentration of *(E)*-2-octenal was therefore 2.602 μg mL^−1^ (SE 1.202). In terms of per unit quantity of dry sebum extract, the concentration of *(E)*-2-octenal was 5.390 (SE 0.512) μg mg^−1^ in donkey 1, 21.514 (SE 0.331) μg mg^−1^ in donkey 2 and 10.290 (SE 0.376) μg mg^−1^ in donkey 3. The average across the donkeys was 12.398 μg mg^−1^ (SE 4.772). The LOD and the LOQ were found to be 0.935 (SE 0.005) and 0.308 (SE 0.002) μg mL^−1^ respectively. The LOQ was smaller than the first point of the calibration curve (<1.135 ng mL^−1^) indicating that, assuming linearity, smaller quantities than the smallest calibrated could be estimated from corresponding chromatographic areas, although for robust estimates from the regression analysis it would still be advisable to remain within the limits of the calibration.

### 3.2 Olfactometer assays

**Fig. 2:**
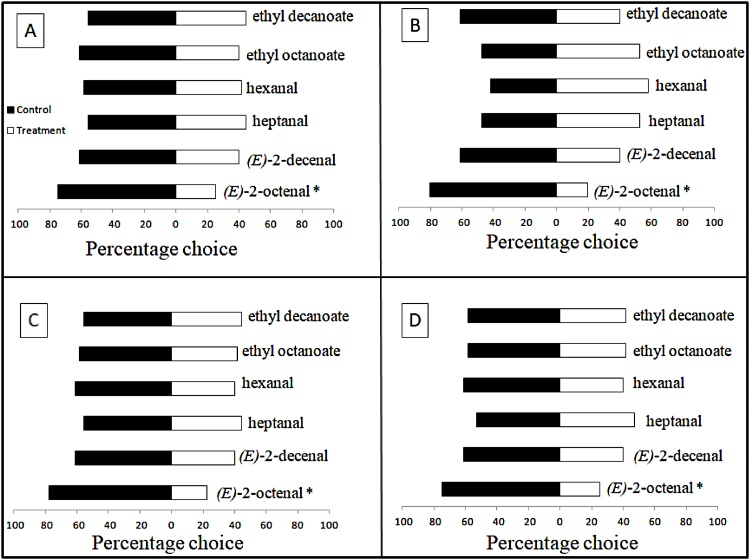
Response of *Amblyomma sculptum* nymphs (*n* = 36) in Y-tube olfactometer assays to five compounds isolated from horses and donkeys and *(E)*-2-octenal tested at four different concentrations 1.0 M (A), 0.50 M (B), 0.25 M (C) and 0.125 M (D). *Significant effect of the chemical using a Chi-square test (p < 0.05) on 1 ° of freedom. Full results from the olfactometer assays, detailing counts of nymphs, Chi-squared statistics and p-values are given in Table S2 (Supplementary Information).

**Fig. 3:**
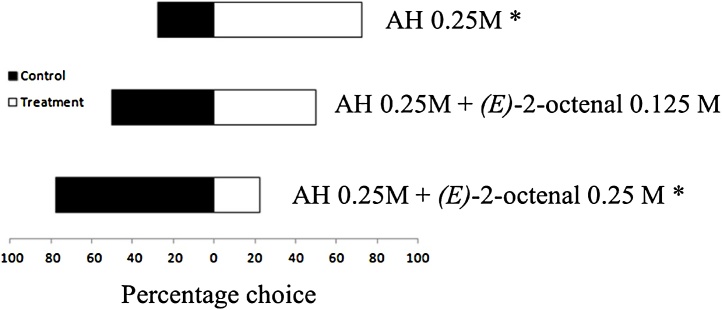
Responses of *Amblyomma sculptum* nymphs (*n* = 36) to an attractant, ammonium hydroxide (AH), and to a mixture of an attractant and a repellent *(E)*-2-octenal in Y-tube olfactometer assays. *Significant effect of the chemical using a Chi-square test (p < 0.05) on 1 ° of freedom. Full results from the olfactometer assays, detailing counts of nymphs, Chi-squared statistics and p-values are given in Table S3 and S4 (Supplementary Information).

Preliminary assays with CO_2_ showed that the equipment was adequate to proceed with the experiments, as 72% of nymphs were attracted to this compound (χ² = 6.25, 1 df; p = 0.01241933). In Y-tube olfactometer assays with *A. sculptum* nymphs, there was no significant response to the solvent control. There was no preference for the arm containing the odour of dry sebum extract, collected from either donkeys or horses, compared to the arm containing the control (data not shown). Furthermore, there was no preference for either donkey odour or horse odour when presented in a dual-choice experiment (data not shown). When hexanal, heptanal, *(E)*-2-decenal and *(E)*-2-octenal were tested *vs.* a solvent control, ticks responded only to (*E*)-2-octenal. Significantly more nymphs preferred the control at a concentration of 1.0 M (χ ² = 8.028, 1 df; p = 0.005), 0.50 M (χ ² = 12.25, 1 df; p < 0.001), 0.25 M (χ ² = 10.028, 1 df; p = 0.002) and 0.125 M (χ ² = 8.028, 1 df; p = 0.005) (Fig. 2). When tested at a concentration of 0.0625 M, no repellency was observed (χ ² = 0.694, 1 df; p = 0.405). In tests with the host-derived attractant ammonia (Fig. 3), addition of (*E*)-2-octenal removed preference of nymphs to the arm containing the attractant, and an increase in (*E*)-2-octenal concentration to 0.25 M resulted in nymphs preferring the control arm.

## 4 Discussion

In this study, we report the first identification of an allomone from a non-host species, in which donkeys are known to be more resistant to *A. sculptum* parasitism than horses. Our results reinforce the hypothesis that vertebrate non-hosts, closely related to host taxa, may produce allomones against ticks, as demonstrated by other authors with ticks, flies and red mites and their respective non-hosts (Bett et al., 2015; Borges et al., 2015; Gikonyo, et al., 2003; Louly, et al. 2010; Oliveira Filho et al., 2017; Pageat, 2005; Saini et al., 2017).

When the dry sebum extracts of horses were tested in behavioural assays, nymphs were not attracted to extracts. It is important to emphasize that it is not always possible to reproduce *in vitro* what is found in nature and *vice versa*, because odour plumes, chemical compounds and their concentrations may differ in the laboratory from that found in the field (Reisenman et al., 2016). Other compounds present in the breath of potential hosts such as 1-octen-3-ol, acetone, nitric oxide and carbon dioxide (CO_2_) are important host location cues for ticks (McMahon and Guerin, 2002). The five compounds found in both host species, ie. hexanal, heptanal, *(E)*-2-decenal, ethyl octanoate and ethyl decanoate neither attracted nor repelled *A. sculptum* nymphs at any tested concentration. Hexanal and heptanal were already reported in the odours of rabbits, ruminants (bovine, giraffe and goat) and sea birds, as well as human sweat (Douglas et al., 2004; Jaleta et al., 2016; Meijerink et al., 2000; Osterkamp et al., 1999; Steullet and Guerin, 1994; Wood and Weldon, 2002). *(E)*-2-Decenal, ethyl octanoate and ethyl decanoate are largely found in meat and dairy products (Condurso et al., 2008; Liu et al., 2004; NCBI, 2017a) and in human sweat (Meijerink et al., 2000). To the best of our knowledge there are no reports of altered tick behaviour to any of these compounds, and no electrophysiological response were reported when *A. sculptum* olfactory sensilla were exposed to heptanal (Soares and Borges, 2012). Further work is needed to confirm whether combinations of these compounds are required for a behavioural response in ticks, similar to that observed by Osterkamp et al. (1999), who showed that a combination of host odours was necessary to promote questing behaviour of *R. microplus* and *Ixodes ricinus*.

When tested in behavioural assays with *A. sculptum* nymphs, the donkey-specific compound (*E*)-2-octenal was repellent at four concentrations tested and interfered with the attractiveness of ammonia, a known volatile host compound. In our previous work, the allomones 2-hexanone and benzaldehyde produced by beagle dogs were repellent against the tick *R. sanguineus* s. l. only at the highest concentration tested (7.8%) (Borges et al., 2015). In this study, the donkey-produced allomone, *(E)*-2-octenal, was repellent at a much lower concentration (0.125 M = 1.25%). These results suggest that allomones from donkeys have a higher efficacy than dog allomones or a greater sensitivity of *A. sculptum* nymphs to this compound. The tick *R. sanguineus* s. l. can be found on several mammal hosts, but has a predilection to parasitize dogs (Dantas-Torres, 2010), whereas *A. sculptum* has a wider specificity range, being found on mammals, reptiles and birds (Barros-Battesti et al., 2006). Allomones against ticks are produced by unsuitable hosts in which tick feeding can lead to impaired tick development (Louly et al., 2009, 2010; Weldon, 2010, 2013). Considering that horses and donkeys can be generally found on the same environment, it is important for *A. sculptum* to use a reliable cue to avoid parasitism on non-host donkeys and so avoid reduced development (Castagnolli et al., 2003).

*(E)*-2-Octenal can be found in volatiles from different species such as cows and goats (Jaleta et al., 2016) and in edible fungi, *Tuber* spp. (Pezizales: Tuberaceae) (Splivallo et al., 2007), but the role of this compound in these organisms is unclear. It is also noteworthy that this compound is the major chemical component of the alarm pheromone for stink bugs and bed bugs, repelling predators and dispersing con-specific individuals (Marques et al., 2007; Noge et al., 2012; Pareja et al., 2007). Similar repellent activity for such insects and ticks can be explained by a conserved evolutionary process from a common ancestor or a co-evolutionary gain (Moore et al., 2006).

From the work described here, *(E*)-2-octenal has the potential to be used as a repellent for reducing *A. sculptum* populations on animal and human hosts, as has been demonstrated for *R. sanguineus* on dog hosts by Oliveira Filho et al. (2017) using benzaldehyde and 2-hexanone. For the latter, a slow release formulation of the allomone caused a reduced load of *R. sanguineus* s. l. on dogs exposed to an artificially infested tick environment. Thus, slow release formulations of *(E*)-2-octenal could be produced and used on capybaras, horses, and even on humans as the compound is considered harmless and used as a flavoring ingredient for cherries, dairy products, nuts and meat (NCBI, 2017b). Besides, reducing *A. sculptum* tick load of capybaras could also reduce prevalence of BSF, as capybaras are the main amplifier for *R. rickettsii* (Labruna, 2009). Our results therefore justify further research for the commercial development of the compound as a new technology for *A. sculptum* control.
