# Supplementary material for: Identification of a non-host semiochemical from tick-resistant donkeys (Equus asinus) against Amblyomma sculptum ticks
Source: Ticks Tick Borne Dis. 2019 Apr;10(3):621–7. doi: 10.1016/j.ttbdis.2019.02.006 (PMC6446183; doi:10.1016/j.ttbdis.2019.02.006)

**SUPPLEMENTARY INFORMATION**

**Fig. S1.** Analytical curve obtained with *(E)*-2-octenal standard solutions (*n*= 18) (A); *(E)-*2-octenal response factor *vs.* concentration of *(E)*-2-octenal standard solution (*n*=18) (B).


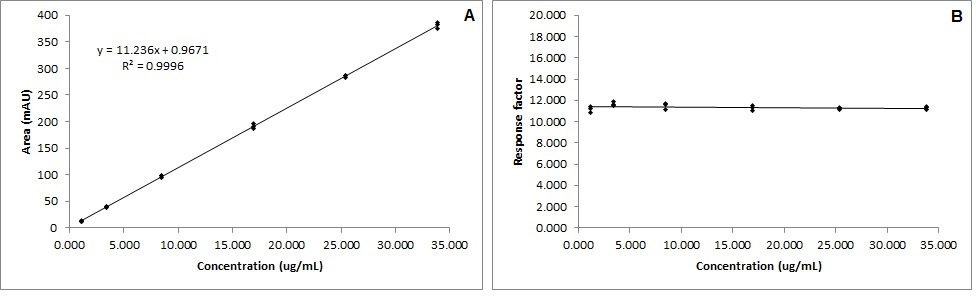


**Table S1** Summary of the output of the ANOVA for the linear regression analysis.

| ANOVA |  |  |  |  |  |  |
| --- | --- | --- | --- | --- | --- | --- |
|  | *df* | *SS* | *MS* | *F* | *Significance F* |  |
| Regression | 1 | 316727.7362 | 316727.7362 | 41166.38001 | 1.01963E-28 |  |
| Residual | 16 | 123.1015158 | 7.693844738 |  |  |  |
| Total | 17 | 316850.8377 |  |  |  |  |
|  |  |  |  |  |  |  |
|  | *Coefficients* | *Standard Error* | *t Stat* | *P-value* | *Lower 95%* | *Upper 95%* |
| Intercept | 0.967144436 | 1.050749015 | 0.92043335 | 0.37101634 | -1.260343969 | 3.19463284 |
| X Variable 1 | 11.23648231 | 0.055380776 | 202.8949975 | 1.01963E-28 | 11.11908031 | 11.35388431 |

**Table S2.** Full results from the olfactometer assays detailing counts of nymphs, Chi-squared statistics, p-values and percentage choice from Y-tube olfactometer assay with compounds found in horses and donkeys.


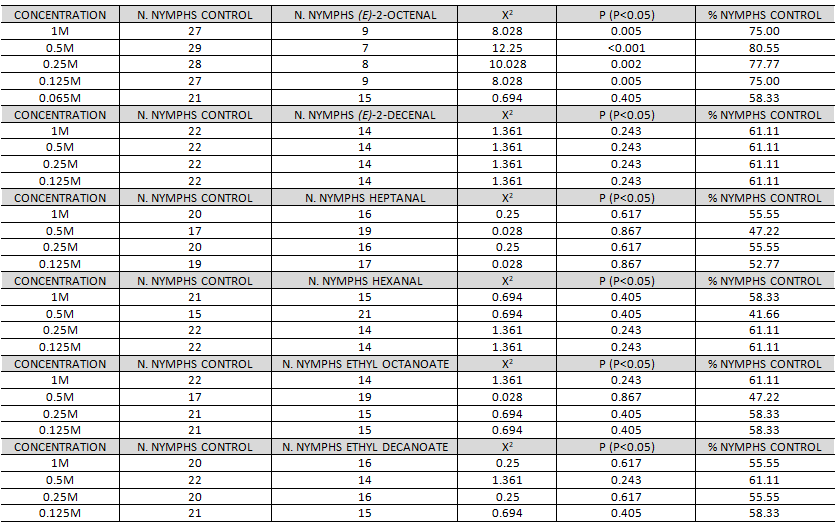


**Table S3.** Full results from the olfactometer assays detailing counts of nymphs, Chi-squared statistics, p-values and percentage choice from Y-tube olfactometer assay with ammonium hydroxide.


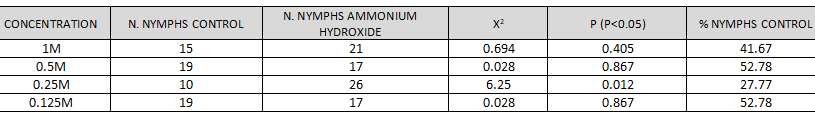


**Table S4.** Full results from the olfactometer assays detailing counts of nymphs, Chi-squared statistics, p-values and percentage choice from Y-tube olfactometer assay with the association between an attractive compound (ammonium hydroxide) and a repellent compound (*E*-2-octenal) to verify a masking effect.


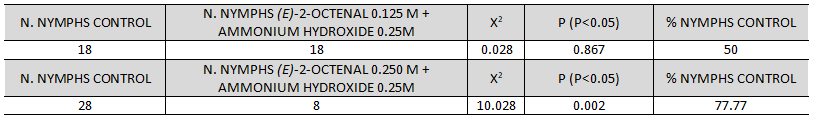

Supplement: Supplementary file 1 [file mmc1.docx]
